# Evaluation of a program for routine implementation of shared decision-making in cancer care: study protocol of a stepped wedge cluster randomized trial

**DOI:** 10.1186/s13012-018-0740-y

**Published:** 2018-03-27

**Authors:** Isabelle Scholl, Pola Hahlweg, Anja Lindig, Carsten Bokemeyer, Anja Coym, Henning Hanken, Volkmar Müller, Ralf Smeets, Isabell Witzel, Levente Kriston, Martin Härter

**Affiliations:** 1 Department of Medical Psychology, University Medical Center Hamburg-Eppendorf, Martinistraße 52, 20246 Hamburg, Germany; 2 II. Department of Medicine, University Medical Center Hamburg-Eppendorf, Martinistraße 52, 20246 Hamburg, Germany; 3 Department of Gynecology, University Medical Center Hamburg-Eppendorf, Martinistraße 52, 20246 Hamburg, Germany; 4 Department of Oral and Maxillofacial Surgery, University Medical Center Hamburg-Eppendorf, Martinistraße 52, 20246 Hamburg, Germany

**Keywords:** Shared decision-making, Implementation science, Cancer, Health services research, Stepped wedge design, Cluster randomized controlled trial

## Abstract

**Background:**

Shared decision-making (SDM) has become increasingly important in health care. However, despite scientific evidence, effective implementation strategies, and a prominent position on the health policy agenda, SDM is not widely implemented in routine practice so far. Therefore, we developed a program for routine implementation of SDM in oncology by conducting an analysis of the current state and a needs assessment in a pilot study based on the Consolidated Framework for Implementation Research (CFIR). Based on these results, the main aim of our current study is to evaluate the process and outcome of this theoretically and empirically grounded multicomponent implementation program designed to foster SDM in routine cancer care.

**Methods:**

We use a stepped wedge design, a variant of the cluster randomized controlled trial. The intervention to be implemented is SDM. Three participating clinics of one comprehensive cancer center will be randomized and receive the multicomponent SDM implementation program in a time-delayed sequence. The program consists of the following strategies: (a) SDM training for health care professionals, (b) individual coaching for physicians, (c) patient activation strategy, (d) provision of patient information material and decision aids, (e) revision of the clinics’ quality management documents, and (f) critical reflection of current organization of multidisciplinary team meetings. We will conduct a mixed methods outcome and process evaluation. The outcome evaluation will consist of four measurement points. The primary outcome is adoption of SDM, measured by the 9-item Shared Decision Making Questionnaire. A range of other implementation outcomes will be assessed (i.e., acceptability, readiness for implementing change, appropriateness, penetration). The implementation process will be evaluated using stakeholder interviews and field notes. This will allow adapting interventions if necessary.

**Discussion:**

This study is the first large study on routine implementation of SDM conducted in German cancer care. We expect to foster implementation of SDM at the enrolled clinics. Insights gained from this study, using a theoretically and empirically grounded approach, can inform other SDM implementation studies and health policy developments, both nationally and internationally.

**Trial registration:**

clinicaltrials.gov, NCT03393351. Registered 8 January 2018.

**Electronic supplementary material:**

The online version of this article (10.1186/s13012-018-0740-y) contains supplementary material, which is available to authorized users.

## Background

Many patients wish to play an active role in their health care [1–5]. Shared decision-making (SDM) is a promising means towards patient participation in health care. SDM is defined as an interactional process in which the patient and the health care professional (HCP) aim to reach medical decisions together, based on shared information and the best available evidence [6, 7]. The HCP supports the patient in weighing the costs and benefits of different options [8]. SDM is especially relevant in oncology, where complex treatment options with varying short- and long-term side effects exist and where the disease and its treatments often have a considerable impact on the patient’s health-related quality of life [9].

For years, national and international health care policies call for SDM implementation. Reasons are among others an ethical imperative for SDM [10], reduction of unwarranted healthcare practice variation [11], reduced use of options not clearly associated with benefits, and increased use of options clearly associated with benefits [12]. However, despite the legal commitment towards SDM in several countries (e.g., Germany [13, 14], the UK [15], the USA [16]), the implementation of SDM in routine care is lacking [3, 4, 17–22].

Research on SDM focused on clinician- or patient-mediated interventions to foster SDM [23]. A Cochrane review [12] showed that a combination of clinician- and patient-mediated interventions is most likely to be successful. As for *clinician-mediated interventions*, SDM trainings can teach HCPs how to involve patients in the decision-making process. Several studies conducted in Germany showed positive effects of training programs such as better SDM skills of physicians and increased patient participation and satisfaction [24–26]. A systematic review concluded that patients need to be informed and empowered in order to be able to play an active role in decision-making [27]. *Patient-mediated interventions* can strengthen the patient’s abilities to engage in the decision-making process. A considerable number of studies showed that patients who learned to use question prompt sheets (i.e., tools that encourage patients to ask their HCPs a range of questions) participated more actively in clinical encounters [28–32]. Patient decision aids (DAs) are another means to increase SDM. These are supportive materials (e.g., brochures) that present different medical options and their costs and benefits to inform patients, and enable them to engage in the decision-making process [33]. There are DAs to be used during the clinical encounter (the so-called encounter decision aids) and others for before or between encounters [8, 33]. A Cochrane review of 105 randomized controlled studies showed positive effects of DAs, e.g., DAs improved the correct perception of benefits and risks of different options, and increased active involvement of patients [34]. The use of DAs in oncology increased knowledge and reduced decisional conflict [35].

There have been efforts to implement SDM in routine practice. A systematic review [36] of implementation studies on DAs concluded that the majority of included studies did not base their design on implementation theory [36]. While this review focused on routine implementation of DAs to foster SDM, there are also SDM implementation projects with multiple implementation strategies to facilitate SDM. Process evaluation of a large-scale multicomponent SDM implementation program in the UK revealed that for successful implementation of SDM in routine care, it was essential to pay attention to the stakeholders’ attitudes, involve all stakeholders at an early stage, and analyze barriers and facilitators [37–39]. This is in line with recommendations from implementation research [40].

Grol et al. [41] emphasize the relevance of theory-based implementation programs to change health care processes, to avoid missing fundamental factors, and to enable transfer and adaptation to other settings. One approach to develop a theory-based implementation program is the Consolidated Framework for Implementation Research (CFIR) by Damschroder et al. [42], a comprehensive framework for routine implementation in the context of health services research. It describes the need of a pre-implementation phase to understand the viewpoints of different stakeholders and develop an empirically based implementation program [42]. Thus, we conducted a pilot study preceding the study at hand that consisted of a thorough analysis of current state and implementation needs. The results of the pilot study are described in detail elsewhere [17, 18, 43–45]. We identified a lack of SDM-specific HCP communication behavior as the main barrier towards the uptake of SDM in the pilot study. The development of our multicomponent implementation program is based on these empirical results as well as the CFIR for theoretical grounding.

## Methods

### Aim

The main aim of this study is to evaluate the process and outcome of a theoretically and empirically grounded multicomponent implementation program designed to foster implementation of shared decision-making into routine clinical practice in cancer care clinics (clinics are the clusters).

### Study design

**Fig. 1:**
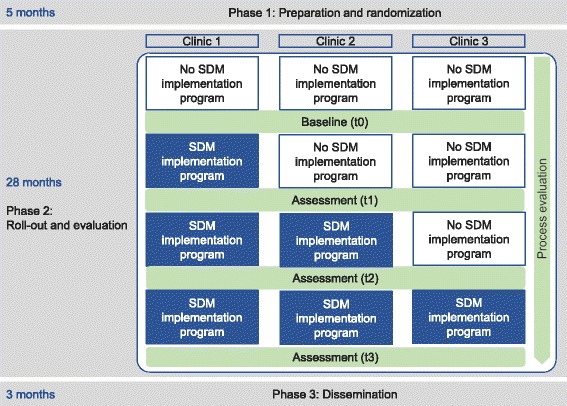
Study design

We consider the implementation program that is to be evaluated as a complex intervention consisting of multiple interacting components, characterized by variability, adaptivity, and interactions with the context [46, 47]. The study uses a stepped wedge design. The stepped wedge design is a variant of the cluster randomized controlled trial. It is increasingly used to evaluate routine implementation of interventions when individual randomization is either not feasible or inappropriate [47, 48], which is the case for our research objective. In the stepped wedge design, participating clinics will receive the multifaceted implementation program in a randomized sequence, as shown in Fig. 1. For preparing this protocol report, we followed CONsolidated Standards of Reporting Trials (CONSORT) extension for Cluster Trials [49] (see Additional file 1), the Standards for Reporting Implementation Studies (StaRI) [50], the Criteria for Reporting the Development and Evaluation of Complex Interventions in Healthcare (revised guideline CReDECI 2) [51], and the Standard Protocol Items for Clinical Trials (SPIRIT) [52], where applicable.

### Setting

The study will be carried out in three clinics at a university hospital’s comprehensive cancer center in Germany: Department of Medical Oncology, Department of Gynecology, and Department of Oral and Maxillofacial Surgery. Conducting the study in three clinics within the same center allows for controlling the influence of outer setting to a certain degree [53]. These clinics were chosen because the heads of department expressed willingness and interest in implementing SDM in their clinics. They consented to participate in the study before randomization. Active promotion of a culture change towards SDM by the heads of department was identified as a key facilitator for the implementation of SDM in the previously conducted pilot study and has also been described in the literature [54].

### Participants

Participants of this study will be both patients and HCPs. Inclusion criteria for participating patients are (a) being diagnosed with a neoplasm (ICD 10: C00-D49), (b) receiving inpatient or outpatient treatment at one of the participating clinics, (c) being at least 18 years of age, and (d) speaking German. Severe cognitive impairment is an exclusion criterion. Participating HCPs are the physicians and nurses working at the respective clinics. No exclusion criteria apply for HCPs.

### Intervention

The intervention to be implemented within this study is SDM, as defined above. The implementation program to foster shared decision-making in routine cancer care consists of multiple components. It is theoretically grounded in the CFIR as well as existing literature on SDM implementation and empirically based on the results of the pilot study mentioned above. It consists of six clinician- and patient-mediated components. Those pertain to the individual participant level, the cluster level, or both. Details can be found below and in Table 1. The components will be delivered as standardized as possible across the participating clinics. All materials and tools used in the different components will use the same study label and slogan (“Behandlungswege gemeinsam entscheiden”, English: deciding treatment paths together).

**Table 1:** Specifications of the six components of the implementation program for SDM, as suggested by Proctor [80]

	SDM training for HCPs	Individual coaching for physicians	Patient activation strategy	Provision of patient information material and decision aids	Revision of the clinic’s quality management documents	Critical reflection of current organization of MDTMs
Actor(s)	Trained HCPs of respective clinic (trained by research team in a train-the-trainer workshop), research team	Research team	Clinic staff and research team	Clinic staff and research team	Research team, quality management department and head HCPs of each clinic	Clinical staff and research team
Action(s)	SDM training for physicians and nurses	Participant observation of physician-patient interaction and provision of feedback	Dissemination of material encouraging patients to ask questions regarding treatment options	Dissemination and use of information material and decision aids	Inclusion of SDM in quality management documents	Meetings with respective head of clinics and members of the clinical teams responsible for the MDTMs
Target(s) of action	HCPs working at respective clinic	HCPs working at respective clinic	Patients being treated in respective clinic	Patients being treated in respective clinic	All staff working at respective clinic	All patient cases discussed in MDTMs
CFIR domain	Individual level and inner setting*	Individual level	Individual level	Individual level	Inner setting*	Inner setting*
Temporality	Beginning of implementation phase in respective clinic	First coaching should be within 4 weeks after training	Throughout implementation phase in respective clinic with start at beginning of phase	Throughout implementation phase in respective clinic with start after HCP training	Beginning of implementation phase in respective clinic	Throughout implementation phase in respective clinic
Dose	2 h training	Two audits with oral and written feedback per HCP	Initial setup of material in different clinic areas, need-based restocking	Initial setup of material in different clinic areas, need-based re-stocking	Short oral presentation of new documents in team meetings, combined with email to staff members	Two to three meetings of approx. 60 min per clinic
Justification	[23, 25, 55, 56, 81] and pilot study [17, 18, 45]	[57] and pilot study [17, 18, 45]	[37, 58] and pilot study [17, 18, 45]	[82] and pilot study [17, 18, 45]	Pilot study [17, 18, 45]	Pilot study [43, 44]

*Inner setting = cluster level

#### SDM trainings for HCPs

The group training for this study is based on the training program developed and evaluated by the research groups around Bieber and Härter (the latter being part of the project group) [23–25] as well as a modified version [55, 56], designed for training interprofessional teams rather than physicians only. Physicians and nurses will be trained. This will take into account the pilot study result that it could be beneficial to better include nurses in the decision-making processes [17, 18, 45]. It uses a train-the-trainer approach, where a small group of HCPs will be trained in SDM skills (step 1) and will then train their colleagues in collaboration with the project team (step 2). To standardize this component, all trainers will adhere to a single training protocol and use the same core set of training materials (e.g., definition and models of SDM, rationale for implementing SDM). However, clinical examples will be tailored to each specific clinic.

#### Individual coaching for physicians

Individual coaching (also known as audit and feedback) will be used to improve physicians’ adoption of SDM practice. In line with current research on this strategy [57], it will be provided more than once, both verbally and written. A member of the research team will visit each physician twice to observe clinical encounters and provide oral and written feedback. The coaching will be delivered after the SDM trainings to support the effect of the training component. It will use a standardized form across all clinics.

#### Patient activation strategy

This patient-mediated strategy consists of the Ask Three Questions program, developed and evaluated by Shepherd and colleagues in Australia [58], and implemented in large-scale implementation programs in the UK and the Netherlands [37, 59]. It consists of three short questions that patients can ask their HCP in order to empower the patients to become more involved in medical decision-making. For this study, the three questions will be translated to German and pilot tested using cognitive interviews with patients. This component will be disseminated via multiple pathways (e.g., flyers, posters, postcards). The dissemination material will use one single design throughout all clinics.

#### Provision of patient information material and decision aids

General disease-related patient information material as well as DAs that are available in German will be compiled by the research team. The material will be made available to patients of the respective clinic via multiple pathways (e.g., in folders that patients’ receive, in waiting rooms, on the clinic website). These materials will be clinic-specific. This strategy will also include informing patients about where to find evidence-based health information online (e.g., on the website of the German Guideline Program in Oncology). The latter is a standardized strategy in all clinics.

#### Revision of the clinics’ quality management documents

In order to facilitate better integration of SDM in these documents, a revision of the quality management documents of the specific clinics is initiated together with the responsible stakeholders from the quality management department and the participating clinics. Across all clinics, such a revision will include a comprehensible definition of SDM and specific recommendations on how to implement SDM and the implications of the current German patients’ rights law will be highlighted. These revisions will be announced to all staff members during the implementation phase in each clinic.

#### Critical reflection of current organization of multidisciplinary team meetings (MDTMs)

In order to integrate SDM better into existing workflows at MDTMs, quality improvement meetings will be held with the leading physicians of clinics and members of the clinical teams responsible for the MDTMs to evaluate and possibly re-organize existing MDTMs. The first meeting will follow the same guideline across all clinics. Subsequent changes will be developed in the process and hence might vary from clinic to clinic.

As described in the CFIR [42], slight adaptations of the implementation components over the course of the study are possible, in order to reflect real-world differences in clinic organization and to take into account the nature of implementation research [60].

### Measures and outcomes

The focus of the evaluation will be on implementation outcomes based on Proctor’s taxonomy [61]. The primary outcome will be the uptake of SDM measured by the patient-reported *9-item Shared Decision Making Questionnaire* (*SDM-Q-9*, [62]). The questionnaire was developed in German, and item development was based on nine process elements characterizing SDM [62]. Items are scored on a 6-point Likert scale, ranging from 0 (“strongly disagree”) to 5 (“strongly agree”). A SDM-Q-9 sum score (ranging from 0 to 100) is calculated with higher values indicating a higher extent of SDM. The SDM-Q-9 showed good psychometric properties [62], was used in a range of studies on SDM [63], and currently exists in 16 languages (www.sdmq9.org). The SDM-Q-9 will be administered at all clinics at baseline, 8 months, 16 months, and 24 months.

Secondary outcomes will be the following: (1) *Uptake of SDM* from external observers’ perspective will be assessed by the German version of the *Observer OPTION*^5^ scale ([64, 65], German version: [66]), using audio-recorded medical encounters. This measure will be administered at all clinics at baseline, 8 months, 16 months, and 24 months. Assessors using the Observer OPTION^5^ will be trained by experienced members of the study team (IS, PH). (2) *Acceptability of SDM* (i.e., perception that SDM is agreeable or satisfactory) from the HCPs’ perspective as measured by an adapted version of the acceptability survey developed by McColl et al. [67]. The measure assesses HCPs’ attitudes towards evidence-based medicine. Three members of the study team (AL, PH, IS) chose those items that were deemed relevant for the research questions of the study at hand. Item wording was adapted to specifically address SDM. This measure will be administered at all clinics at baseline, 8 months, 16 months, and 24 months. (3) *Organizational readiness for implementing change* from the HCPs’ perspective as measured by an adapted version of the *Organizational Readiness for Implementing Change* (*ORIC*, [68]) measure. The measure assesses two facets: (1) change commitment (i.e., organizational members’ shared resolve to implement SDM) and (2) change efficacy (i.e., organizational members’ shared belief in their collective capability to implement SDM) [68]. This measure will be administered analogously to the SDM acceptability measure. (4) *Appropriateness of SDM* (i.e., perceived fit, relevance, or compatibility of SDM for the given practice setting) from the HCPs’ perspective as measured by an adapted version of the *IcanSDM* measure [Coudert L, Carmichael P-H, Renaud J-S, Légaré F, Witteman, H. Martineau B, Kröger E, et al. Validation of the IcanSDM instrument to assess clinicians’ perceptions of their ability to adopt shared decision making [in preparation]]. The IcanSDM measures HCPs’ perceived ability to adopt SDM. This measure will be administered analogously to the SDM acceptability measure. (5) *Penetration of SDM at the clinic level* will be assessed using routine data from patient experience surveys of the clinics. Those surveys are administered every 3 years at the clinics and include questions on patients’ experience of the decision-making process. Data from July 2017 (i.e., before the beginning of this study) and data from 3 years into the study (approximately July 2020) will be compared. (6) *Penetration of SDM in MDTMs* as measured by an adapted version of the *Metric for the Observation of Decision Making in Multidisciplinary Team Meetings* (*MDT-MODe*, [69]). The MDT-MODe assesses the quality of the clinical treatment recommendation process in MDTMs (including the quality of different areas of information presented and the quality of team behavior). MDTMs will be observed and evaluated using this measure at all clinics at baseline, 8 months, 16 months, and 24 months.

Additionally, basic demographic information will be assessed from study participants.

### Data collection

A mixed methods evaluation, which has been described as particularly suitable in implementation research [60], will be carried out. It will include qualitative and quantitative formative and summative evaluation steps.

#### Preparation phase

During the preparation phase, cognitive interviews to assess comprehensibility of translated measures and interventions will be conducted with *N* = 10–12 patients and *N* = 10–12 HCPs. Patients will be recruited through the collaborating clinics. HCPs will be recruited at the university medical center. HCPs participating in cognitive interviews will not be those working at the three clinics receiving the implementation program.

#### Allocation

The methodologist of the research team (LK) will generate the randomized allocation sequence for the participating clinics (the units of randomization) using computer-generated random numbers. Enrollment of the clusters (done by IS) will be completed before randomization (allocation sequence concealed). Randomization will be open label due to the sequential nature of the extensive implementation program that makes blinding of clinics and HCPs within clinics unfeasible. Patients are blinded.

#### Outcome evaluation

The outcome evaluation will consist of four measurement points (i.e., baseline, 8 months, 16 months, 24 months). Each measurement point will consist of an assessment phase of 2 months. Due to the design of this study, different samples of individual patients will contribute to each measurement point (no follow-up assessment: independent samples). Clinical staff working at the clinics at the time of data collection will contribute to each measurement point (combination of new and previously surveyed participants with follow-up assessments: partly dependent samples). Patients will be recruited consecutively in the three clinics at all measurement points by a researcher of the study team. They will be approached individually (e.g., after registration at the front desk for outpatients, in wards for inpatients). Patients can participate in this study in three ways: (a) filling in the SDM-Q-9 after the encounter with the HCP, (b) agreeing to the encounter being audio recorded in order to be assessed using the Observer OPTION^5^ measure, or (c) both. The researcher will ask each patient for informed consent. The measures addressing the HCPs’ perspective will be administered in each clinic directly before the intervention program starts and at all consecutive measurement points (i.e., baseline, 8 months, 16 months, and 24 months for clinic 1; 8 months, 16 months, and 24 months for clinic 2; 16 months, and 24 months for clinic 3). All HCPs working at the respective clinic will be invited to fill in the questionnaires (complete enumeration).

#### Process evaluation

The process evaluation has two main purposes: (1) to understand the possible effects of the implementation program and (2) to allow adapting interventions, if necessary (e.g., due to limited use of implementation components). As described by Damschroder [42], the CFIR will be used to guide the process evaluation, aided by additional recommendations [70, 71].

The process of implementation will be evaluated using mainly qualitative research methods through collection of (a) field notes, (b) interview data, and (c) documentary data. We will generate qualitative *field notes* [72], capturing observations (e.g., interactions with HCPs and other staff) made when present in the clinics (e.g., during data collection and delivery of implementation strategies). Furthermore, to assess the experience and response to the implementation program, we will conduct short *interviews* with different HCPs throughout the study, using a purposive sampling strategy with maximum variation approach (e.g., different professions, different hierarchy levels). Interviews will be audio-recorded. We will also *document* how the different implementation strategies are actually delivered to each cluster by continuously writing short reports about the delivery of each strategy in each clinic. By doing so, we will gain insight into the fidelity and reach the implementation program is administered with. Documentation will include internal facilitators and barriers potentially influencing the delivery of the implementation components. In terms of reach, we will monitor who and how many participated in the implementation components. Also, external conditions or factors occurring during the study that might have influenced the delivery of the implementation components will be monitored.

### Handling of potential adverse events and study risks

Due to the empirical evidence for positive effects of SDM for patients (cp. background), no adverse events for patients are expected. The assessments including the SDM-Q-9 and/or audio-recordings of medical encounters are only minimally disruptive. Investment of participating HCPs will be in the form of participation in trainings and coaching. Potentially, through those HCPs will be stimulated to reflect their own work culture and processes. However, we expect the implementation program to increase HCP satisfaction with decision-making rather than decrease it. The planned process evaluation will ensure that potential adverse events will be detected promptly and responded to.

Assessment phases were planned in close collaboration with the participating clinics. Nevertheless, patient flows might fluctuate between months. In case of difficulties reaching the planned sample size, it would be possible to extend data collection phases in order to reach acceptable sample sizes.

### Data analysis

#### Sample size calculation

We aim to identify a small to moderate effect of the intervention on the patients’ assessment of experienced SDM (Cohen’s *d* of 0.3). Although the existing empirical findings are subject to uncertainty, this effect seems both practically relevant and realistic [12]. To identify this effect in a two-sided individual-level parallel group trial with an alpha error probability of 0.05 and a power of 0.80, a total sample of 352 participants would be needed. If an intra-class correlation coefficient of 0.05 is expected and three clinics are measured at four time points each (resulting in a total of 12 clusters; see Fig. 1), around 100 participants per cluster are necessary to maintain the specified alpha and beta error levels of 0.05 and 0.20, respectively [73, 74]. In order to be able to deal flexibly with frequently encountered issues (e.g., unequal-sized clusters, incomplete participant responses), we aim to recruit 360 participants at each time point across all clinics and 1440 participants in total.

As part of the process evaluation, we aim to conduct approx. *N* = 8–15 interviews with different HCPs per clinic. Participant observation will be conducted bi-weekly for 2 to 4 h per clinic. These are estimates and final sample sizes for qualitative data collection will be informed by theoretical saturation.

#### Analysis plan

##### Phase 1

The audio-recordings of cognitive interviews in phase 1 will be transcribed and imported into MAXQDA software (VERBI GmbH, Berlin, Germany). They will be analyzed drawing on the principles of content analysis [75].

##### Phase 2

Data will be entered by members of the research team consecutively, checked for range of data values and possible inconsistencies, and stored at secured servers of the department of the research team. Data will be analyzed using a mixed linear model with a random effect for clinic, a fixed effect for the time interval, and a fixed effect for the intervention effect [76]. An additional analysis will be performed to test for a possible interaction between intervention and time interval. If participant characteristics vary across clinics/time intervals, they will be used as participant-level covariates in the analysis model. In the case that a random effects model is not estimable (e.g., non-convergence due to the relatively low number of clinics), a fixed effects model will be used. Missing data patterns will be analyzed and dealt with adequately (e.g., by using maximum likelihood estimation). Two-sided testing will be applied throughout, and findings with an alpha error rate below 0.05 will be considered statistically significant.

##### Process evaluation

The audio-recordings of HCP interviews will be transcribed. Transcripts of interviews, field notes, and documentary data will be imported into MAXQDA software (VERBI GmbH, Berlin, Germany) and will be analyzed drawing on the principles of content analysis [75].

### Data monitoring

The research team will meet once weekly during the entire duration of the study for executive as well as quality management purposes. An international study advisory board with six members from Germany, the USA, and Australia that cover different aspects of scientific expertise beneficial to the project will meet in the form of conference calls (60 to 90 min) approximately twice a year. Prior to each meeting, members of the advisory board will receive a short written summary on the current stage of the study and topics to be discussed during the meeting. The advisory board is independent from the sponsor of this study.

## Discussion

This study is the first of its kind to focus on implementing SDM in routine cancer care in Germany. Other health care institutions (e.g., comprehensive cancer centers) and implementation researchers can use the results of this study to inform future large-scale implementation programs on SDM. The results could also be interesting for health policy activities, specifically the German National Cancer Plan, which also calls for a better implementation of SDM in cancer care [77].

In case of successful adoption of SDM through the proposed program, further research could also assess other, more distant, implementation outcomes, such as implementation costs (i.e., the cost impact of implementation efforts) and sustainability (i.e., the extent to which SDM is maintained or institutionalized within a service setting’s on-going, stable operations) [61]. This could be done in a follow-up study.

## Dissemination

The results of the project will be published in scientific journals, possibly in open-access format to ensure high accessibility to the research and the clinical community as well as other interested individuals and institutions. Results will also be presented at national and international conferences. Social media will be used for dissemination of results (e.g., via Twitter accounts of the research group and the international collaboration partners). Furthermore, the material of the different implementation strategies described above will be made available either as online open-access appendices to the publications or for download and free use through the well-known German website on shared decision-making hosted by the research group (www.patient-als-partner.de).

## Additional file

Additional file 1: CONSORT extension for cluster trials checklist. (DOCX 28 kb)

## Abbreviations

AC: Anja Coym
AL: Anja Lindig
CB: Carsten Bokemeyer
CFIR: Consolidated Framework for Implementation Research
DAs: Decision aids
HCP: Health care professional
HH: Henning Hanken
IQWIG: Institute for Quality and Efficiency in Health Care
IS: Isabelle Scholl
IW: Isabell Witzel
LK: Levente Kriston
MDTM: Multidisciplinary team meeting
MDT-MODe: Metric for the Observation of Decision Making in Multidisciplinary Team Meetings
MH: Martin Härter
ORIC: Organizational Readiness for Implementing Change
PH: Pola Hahlweg
RS: Ralf Smeets
SDM: Shared decision-making
SDM-Q-9: 9-Item Shared Decision Making Questionnaire
VM: Volkmar Müller
